# Proteomic analysis of seed storage proteins in wild rice species of the *Oryza* genus

**DOI:** 10.1186/s12953-014-0051-4

**Published:** 2014-11-30

**Authors:** Chunmiao Jiang, Zaiquan Cheng, Cheng Zhang, Tengqiong Yu, Qiaofang Zhong, J Qingxi Shen, Xingqi Huang

**Affiliations:** College of Life Science, Yunnan University, Kunming, Yunnan 650031 P.R. China; Biotechnology & Genetic Germplasm Institute, Yunnan Academy of Agricultural Sciences, Kunming, Yunnan 650223 P.R. China; School of Life Science, University of Nevada, Las vegas, USA

**Keywords:** Wild rice species, Seed storage proteins, Glutelin acidic subunits, 2-DE, LC/ESI-MS/MS, Nutritional quality

## Abstract

**Background:**

The total protein contents of rice seeds are significantly higher in the three wild rice species (*Oryza rufipogon* Grill., *Oryza officinalis* Wall. and *Oryza meyeriana* Baill.) than in the cultivated rice (*Oryza sativa* L.). However, there is still no report regarding a systematic proteomic analysis of seed proteins in the wild rice species. Also, the relationship between the contents of seed total proteins and rice nutritional quality has not been thoroughly investigated.

**Results:**

The total seed protein contents, especially the glutelin contents, of the three wild rice species were higher than those of the two cultivated rice materials. Based on the protein banding patterns of SDS-PAGE, *O. rufipogon* was similar to the two cultivated rice materials, followed by *O. officinalis*, while *O. meyeriana* exhibited notable differences. Interestingly, *O. meyeriana* had high contents of glutelin and low contents of prolamine, and lacked 26 kDa globulin band and appeared a new 28 kDa protein band. However, for *O. officinali* a 16 kDa protein band was absent and a row of unique 32 kDa proteins appeared. In addition, we found that 13 kDa prolamine band disappeared while special 14 kDa and 12 kDa protein bands were present in *O. officinalis*. Two-dimensional gel electrophoresis (2-DE) analysis revealed remarkable differences in protein profiles of the wild rice species and the two cultivated rice materials. Also, the numbers of detected protein spots of the three wild rice species were significantly higher than those of two cultivated rice. A total of 35 differential protein spots were found for glutelin acidic subunits, glutelin precursors and glutelin basic subunits in wild rice species. Among those, 18 protein spots were specific and 17 major spots were elevated. Six differential protein spots for glutelin acidic subunits were identified, including a glutelin type-A 2 precursor and five hypothetical proteins.

**Conclusion:**

This was the first report on proteomic analysis of the three wild rice species. Overall results suggest that there were many new types of glutelin subunits and precursor in the three wild rice species. Hence, wild rice species are important genetic resources for improving nutritional quality to rice.

**Electronic supplementary material:**

The online version of this article (doi:10.1186/s12953-014-0051-4) contains supplementary material, which is available to authorized users.

## Background

Rice (*Oryza sativa* L.) is one of the world’s most important staple crops and more than half of the population depends on it as a main source of nutrition. Consequently, rice grain is a primary source of proteins for humans, particularly in Asia. Compared with other cereals, rice has a relatively low protein contents ranging from 7% to 10% of the grain dry weight [1]. The nutritional value of rice could thus be raised by increasing its protein contents. Rice seed storage proteins are generally divided into water-soluble albumins, salt-soluble globulins, alcohol-soluble prolamines, and alkali or acid soluble glutelins [2]. In most cereals, prolamines are the major seed storage proteins, whereas in rice grains glutelin is the major protein of the starchy endosperm and accounting for 60% to 80% of total seed protein. In contrast, prolamine accounts for 20% to 30% [3]. During rice seed development, rice glutelins are first synthesized as precursors of 57 kDa proglutelins that were processed prototypically into acidic (*α*) and basic (*β*) subunits, which molecular weights of 22 to 23 kDa and 37 to 39 kDa, respectively [4,5]. Rice seed proteins localize in two types of protein bodies (PB), PB-I and PB-II. PB-I contains prolamines, whereas PB-II is rich in glutelins and globulins [4,6]. PB-I is less digestible than PB-II suggesting that prolamine in PB-I is nutritionally less meaningful for human than glutelin in PB-II. Therefore, the nutritional value of rice could be enhanced by increasing the glutelin and globulin contents [7].

In recent years, enhancing rice seed storage proteins to improve rice nutritive value has gradually become one of the important targets for rice quality breeding. Protein contents and composition are crucial to rice grain quality and nutritional value [8]. Genetic base of rice seed proteins in current rice varieties is relatively narrow. It is necessary to explore new superior genetic resources for further nutritional improvement. Wild relatives of rice are important reservoirs for useful genes and not only have provided crops with resistance to pets and disease, enhanced tolerance to biotic or abiotic stresses but also are presumed to contain promising genetic resources for quality improvement [9]. In addition to cultivated rice (*Oryza sativa* L.), there are three wild rice species, namely *Oryza rufipogon* Griff., *Oryza officinalis* Wall. and *Oryza meyeriana* Baill., in the Yunnan province, China. Previous studies have shown that total seed protein contents in husked seeds of the three wild rice species were significantly higher than that of cultivated rice [10]. However, there is no report on systematic proteomic analyses on total seed proteins and the components of these proteins in the three wild rice species. Proteomics is a tool that can be used both to visualize and compare complex mixtures of proteins and to gain insights on the individual proteins involved in specific biological responses [11]. Xie et al. [12] analyzed and compared the embryo protein spots among the three cultivated rice varieties (9311, PA64S and LYP9) by two-dimensional gel electrophoresis (2-DE). Komatsu et al. analyzed the protein of rice embryo, endosperm and leaf by 2-DE, and detected 600, 100 and 150 protein spots, respectively [13]. In this study, we reported a proteomic analysis of proteins extracted from dehulled seeds of the three wild rice species and two cultivated rice materials with 2-DE map. The main objective of this study was to identify the differentially expressed seed proteins between the wild rice species and cultivated rice and to reveal the causes of the significant differences. To our knowledge, this was the first high quality 2-DE maps of seed storage proteins from the three wild rice species in the Yunnan province.

## Results

### The difference of the total seed protein contents between the wild rice species and cultivated rice was largely due to different glutelin contents

**Figure 1:**
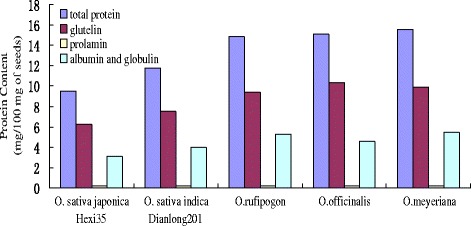
**Total protein and glutelin contents were higher in wild rice species than in the cultivated rice.**

The total seeds protein contents of the three wild rice species were higher than the two cultivated rice materials (Figure 1). The total protein contents of *O. rufipogon*, *O. officinalis* and *O. meyeriana* in husked seeds were 14.87 mg/100 mg, 15.12 mg/100 mg and 15.56 mg/100 mg dry seed weight, respectively. In contrast, the values for *O. sativa japonica* Hexi35 and *O. sativa indica* Dianlong201 were only 9.523 mg/100 mg and 11.76 mg/100 mg, respectively (Table 1).

**Table 1:** **The seed protein contents in three wild rice species and two cultivated rice**

**Materials**	**Total protein**	**Glutelin**	**Prolamine**	**Albumin and globulin**
*O. sativa japonica* Hexi35	9.52	6.225	0.212	3.083
*O. sativa indica* Dianlong201	11.76	7.523	0.223	4.014
*O.rufipogon*	14.87	9.384	0.224	5.262
*O.officinalis*	15.12	10.345	0.212	4.563
*O.meyeriana*	15.56	9.893	0.225	5.442

Protein Content (mg/100 mg of seeds).

The glutelin contents between the three wild rice species and two cultivated rice materials were also significantly different. *O. officinalis* contains the highest amount of glutelin contents, whereas *O. sativa japonica* Hexi35 has the lowest glutelin contents (Figure 1, Table 1). Our results suggest that the significant differences of the total protein contents between the wild rice species and cultivated rice materials were mainly due to their different glutelin contents.

### The protein banding patterns of the wild rice species were different from those of cultivated rice

**Figure 2:**
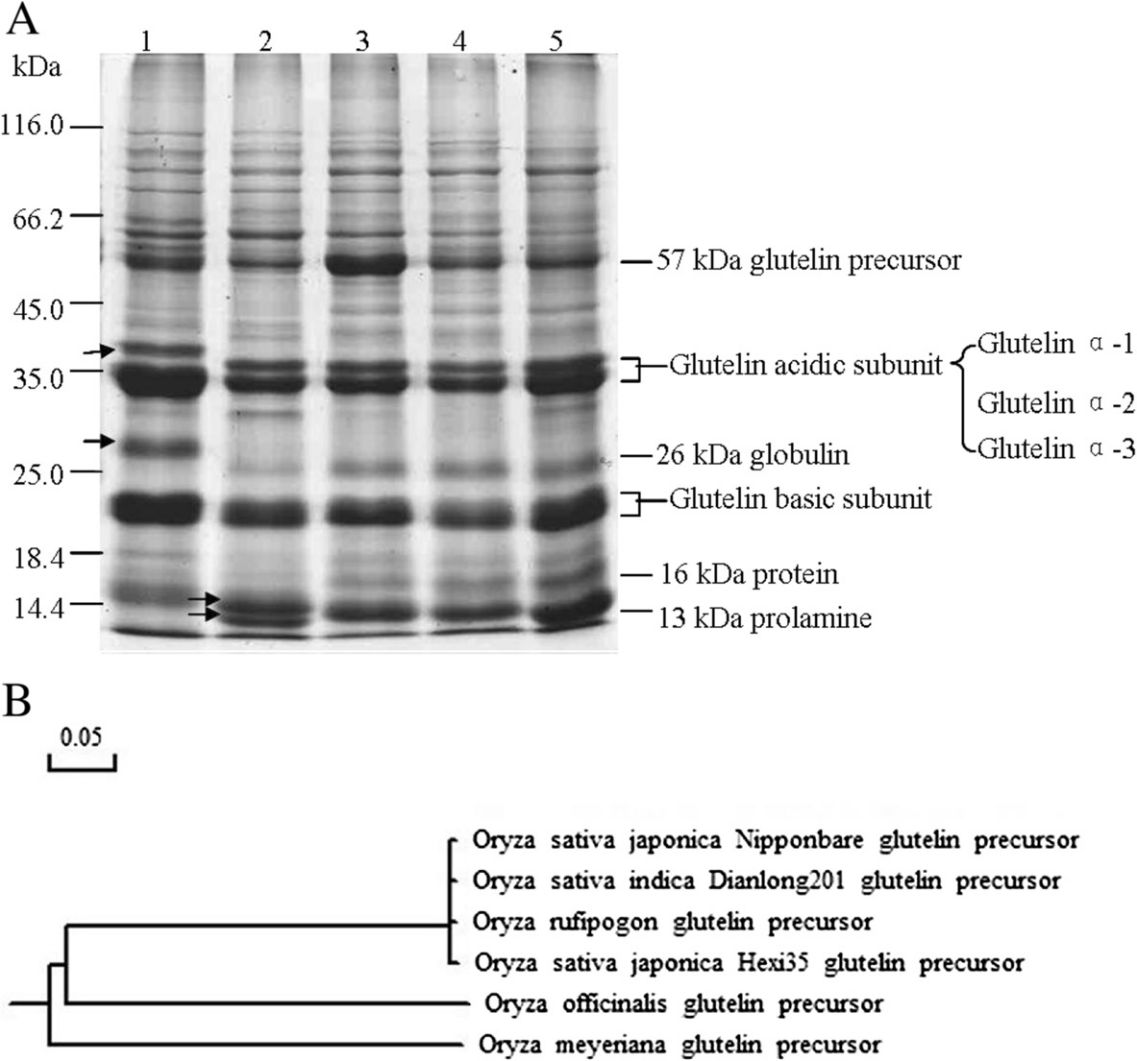
**The SDS-PAGE analysis of five materials and the evolution dendragram of five materials using glutelin precursor DNA. A**: The banding pattern of total seed proteins from *O. meyeriana* is noticeably different from those from other four samples. SDS-PAGE analyses of seed proteins in *O. meyeriana* (lane 1), *O. officinalis* (lane 2), *O. rufipogon* (lane 3), *O. sativa indica* Dianlong201 (lane 4) and *O. sativa japonica* Hexi35 (lane 5). **B**: The evolution dendragram of five materials using glutelin precursor DNA sequence alignment.

There were differences in protein banding patterns between the wild rice species and cultivated rice materials. As shown in Figure 2A, similar numbers of total protein bands were for *O. meyeriana*, *O. officinalis*, *O. rufipogon*, *O. sativa indica* Dianlong201 and *O. sativa japonica* Hexi35, i.e, 16, 17, 18, 19 and 17, respectively. However, marked differences in their protein banding patterns were observed. Among the three wild rice species, the protein patterns of *O. rufipogon* were similar to those of the two cultivated rice, followed by that of *O. officinalis*. However, *O. meyeriana* exhibited notable differences. Compared with cultivated rice, the expression of 57 kDa glutelin precursor was higher in *O. rufipogon* and *O. meyeriana* (Figure 2A).

There were also significant differences in the composition of glutelin acidic subunits between *O. meyeriana* and other materials. The comparison of glutelin acidic subunits in Figure 2A revealed that *α*-1 subunit of glutelin acidic subunits was absent in *O. meyeriana*, while a new protein band of about 40 kDa appeared (as indicated by the arrows in Figure 2A, lane 1). In addition, the expression of glutelin *α*-2 and *α*-3 subunits was much higher than those of the rest of the materials.

Regarding the protein banding patterns of globulin and prolamine, wild rice species were also different from cultivated rice. In *O. meyeriana*, the 26 kDa globulin band was absent, and a new 28 kDa protein band appeared (as indicated by the arrows in Figure 2A, lane 1). In *O. officinalis*, the expression of the 26 kDa globulin was apparently reduced, and the 16 kDa protein band was absent. In addition, we found that the 13 kDa prolamine band disappeared, while special 14 kDa and 12 kDa protein bands were present in *O. officinalis*, as indicated by the arrows in Figure 2A, lane 2.

Analyses of the DNA coding sequence for the glutelin precursor revealed their phylogenetic relationship (Figure 2B). The glutelin precursors of the three cultivated rice and *O. rufipogon* were highly homologous to each other, suggesting their late divergence in evolution. The tree also suggests an early divergence of *O. officinalis* and *O. rufipogon*/cultivated rice and an early divergence of this clade from *O. meyeriana.*

Overall the results of comparing the SDS-PAGE between the three wild rice species and two cultivated rice materials, the protein banding patterns of grutelin acidic subunits and prolamine were significantly different in *O. meyeriana* and *O. officinalis*. The appearing new protein bands and different bands in *O. meyeriana* and *O. officinalis* maybe represented new types of proteins. In the same time, the decrease and absent of prolamine in *O. meyeriana* and *O. officinalis*, which indicated that they are wild rice species of high glutelin and low prolamine.

### The 2-DE analyses of total seed storage proteins of three wide rice species

To further analyze and characterize the differences in protein profiles between the wild rice species and two cultivated rice materials, total seed proteins of five materials were analyzed by 2-DE. The positions of individual proteins on the gels were evaluated with ImageMaster 2D platinum software. A total 338, 344, 458, 300 and 258 protein spots were reproducibly detected for *O. rufipogon*, *O. officinalis*, *O. meyeriana*, *O. sativa indica* Dianlong201 and *O. sativa japonica* Hexi35, respectively (Table 2). Clearly, more protein spots were detected in the three wild rice species than in the cultivated rice materials. The greatest number of spots was detected for *O. meyeriana*; however, least number of spots was detected in *O. sativa japonica* Hexi35. These results suggest that wild rice species may contain more kinds of proteins than cultivated rice.

**Table 2:** **The protein spot numbers were detected from 2-DE of five rice materials by ImageMaster 2D platinum software**

**Protein spot numbers**	***O. rufipogon***	***O. officinalis***	***O. meyeriana***	***O. sativa japonica*** **Hexi35**	***O. sativa indica*** **Dianlong201**
Total protein spots	338	344	458	258	300
^*^I,glutelin acidic subunit	119	144	174	91	125

^*^Glutelin acidic subunit region is mainly concentrated between the 45 kDa-26 kDa on gel maps, and glutelin has the highest expression in this region on 2-DE, therefore the numbers of protein spots in this region among five materials were compared.

Comparison 2-DE maps also revealed differences in the protein profiles of five materials. In general, the protein profiles of *O. rufipogon* showed a high similarity to those of *O. sativa japonica* Hexi35 and *O. sativa indica* Dianlong201. The protein profiles of *O. officinalis* and *O. meyeriana* (especially of *O. meyeriana)* displayed significant differences from those of cultivated rice. In addition, the protein spots for *O. meyeriana* appeared to be darker than those of the rest of the materials, arguing that its protein content was more abundant, consistent with the result shown in Figure 2A.

Meanwhile, through ImageMaster 2D platinum software analysis, we found that the matching protein spots between the *O. rufipogon* and cultivated rice were the most, while the matching protein spots between *O. meyeriana* and cultivated rice were the fewest (Table 3). In the glutelin subunits, the matching rate between the *O. rufipogon* and cultivated rice was also the highest, and the matching rate between the *O. meyeriana* and cultivated rice was the lowest (Table 3). These suggest that *O. rufipogon* is similar to cultivated rice, while there is significantly different between *O. meyeriana* and cultivated rice.

**Table 3:** **The matching protein spots on 2-DE map between Yunnan wild rice species and two cultivated rice materials**

**The matching protein spots number**		***O. sativa japonica*** **Hexi 35**	***O. sativa indica*** **Dianlong 201**	***O. rufipogon***
*O. rufipogon*	Total protein spots matching number	165 pairs	151 pairs	**___**
	Protein spots matching number in glutelin regions	56 pairs,12 protein spots up-regulated	71 pairs, 33 protein spots up-regulated	**___**
*O. officinalis*	Total protein spots matching number	87 pairs	89 pairs	77 pairs
	Protein spots matching number in glutelin regions	42 pairs, 33 protein spots up-regulated	38 pairs, 21 protein spots up-regulated	46 pairs, 24 protein spots up-regulated
*O.meyeriana*	Total protein spots matching number	80 pairs	76 pairs	81 pairs
	Protein spots matching number in glutelin regions	36 pairs,12 protein spots up-regulated	34 pairs, 11 protein spots up-regulated	37 pairs,10 protein spots up-regulated

**Figure 3:**
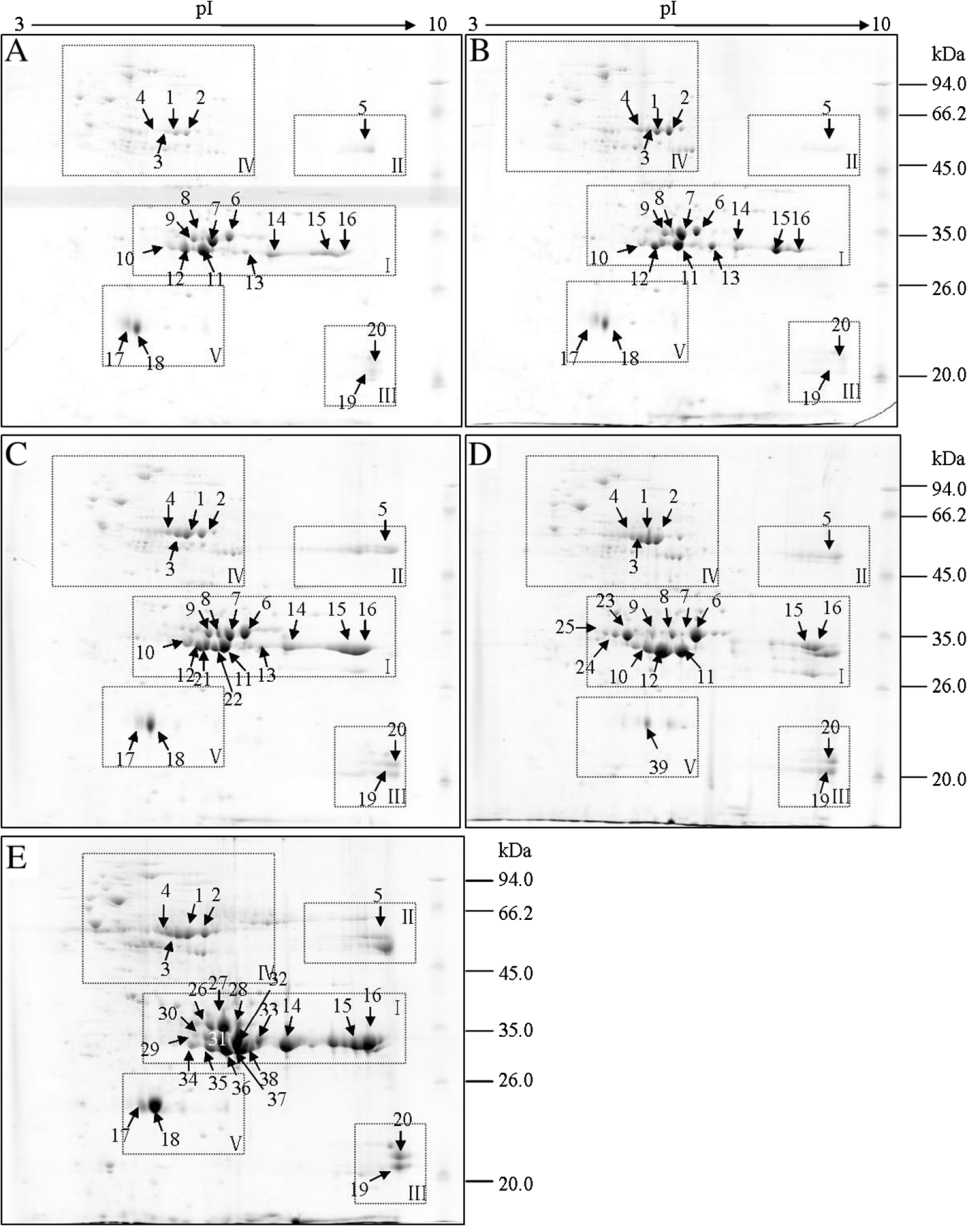
**Representative 2-DE maps of rice total seed storage proteins.** Proteins were extracted from seeds of five rice materials, separated by 2-DE and stained with CBB. The rectangles indicate the regions with differential proteins. The compared protein spots were indicated with corresponding numbers and arrows. **(A)** *O. sativa japonica* Hexi35; **(B)** *O. sativa indica* Dianlong201; **(C)** *O. rufipogon*; **(D)** *O. officinalis*; **(E)** *O. meyeriana.*

The differential protein profiles among five materials were positioned largely in five specific regions of the gels as follows. I, glutelin acidic subunits; II, glutelin precursors; III, glutelin basic subunits; IV, water-soluble proteins; V, globulins, which corresponded to the framed I, II, III, IV and V in Figure 3, and were enlarged in Additional files 1, 2, 3, 4 and 5: Figures S1-S5 to show more details.

### There were some new protein spots in glutelin acidic subunits (I) in wild rice species

The molecular weight of proteins in glutelin acidic subunits was from 26 kDa to 45 kDa and p*I* was from 5.0 to 10.0. Protein spots in glutelin acidic subunits on each global 2-DE map had the highest expression, and obvious differences in the region were observed among five maps. There were significantly different between protein profiles of wild rice species and cultivated rice materials in glutelin acidic subunits (Additional file 1: Figure S1). Especially of *O. officinalis* and *O. meyeriana*, the expression of glutelin in this area was significantly higher than those of the rest of materials, and there existed a lot of differential protein spots. The special differences of protein profiles among five materials in glutelin acidic subunits were as follow:

- *The differences of glutelin acidic subunits in O. rufipogon compared with two cultivated rice materials*: Six protein spots showed increased expression amount in *O. rufipogon* (Additional file 1: Figure S1C), including protein spot 9 (p*I* 6.71, 34 kDa), spot 8 (p*I* 6.88, 34 kDa), spot 6 (p*I* 7.36, 35 kDa), spot 10 (p*I* 6.30, 35 kDa), spot 12 (p*I* 6.54, 34 kDa) and spot 14 (p*I* 8.11, 34 kDa). Additionally, two unique protein spots 21 (p*I* 6.68,34 kDa) and 22 (p*I* 6.83,34 kDa) were detected in *O. rufipogon* (Additional file 1: Figure S1C). Although these two protein spots were detected in *O. sativa indica* Dianlong201 (Additional file 1: Figure S1B), the expression of them were significantly lower than that of *O. rufipogon*, which indicated the expression of glutelin acidic subunits was higher in *O. rufipogon* compared to the two cultivated rice materials.
- *The differences in glutelin acidic subunits of O. officinalis compared with other materials:* Protein spot 7, spot 13 and spot 14 were absent. There were three endemic protein spots in glutelin acidic subunits of *O. officinalis*, including spot 25 (p*I* 5.54, 34 kDa), spot 24 (p*I* 5.72, 34 kDa), spot 23 (p*I* 5.95, 34 kDa) (Additional file 1: Figure S1D). In addition, a row of unique 32 kDa proteins appeared in *O. officinalis* (as shown with an arrow in Additional file 1: Figure S1D). Compared with cultivated rice, the expression of protein spot 6 (p*I* 7.36, 35 kDa), spot 10 (p*I* 6.32, 33 kDa) and spot 12 (p*I* 6.58, 31 kDa) increased.
- *The differences in glutelin acidic subunits of O. meyeriana compared with other materials:* Among five materials, protein spots from *O. meyeriana* in glutelin acidic subunits were very complex and completely different from those of other materials (Additional file 1: Figure S1E). Protein spots in this area can be approximately arranged into three rows: molecular weight with 37 kDa: spot 26 (p*I* 6.65, 37 kDa), spot 27 (p*I* 6.90, 37 kDa) and spot 28(p*I* 7.22, 37 kDa); molecular weight with 35-34 kDa: spot 29 (p*I* 6.55, 35 kDa), spot 30 (p*I* 6.66, 35 kDa), spot 31 (p*I* 6.86, 34 kDa), spot 32 (p*I* 7.13, 34 kDa) and spot 33 (p*I* 7.50, 34 kDa); molecular weight with 33-31 kDa: spot 34 (p*I* 6.64, 33 kDa), spot 35 (p*I* 6.96, 32 kDa), spot 36 (p*I* 7.12, 31 kDa), spot 37 (p*I* 7.18, 31 kDa) and spot 38 (p*I* 7.39, 32 kDa). Whereas for other materials, protein spots in this area only can be roughly arranged into two rows, and the protein spot numbers were obviously less than *O. meyeriana*. In addition, the expression of protein spot 14 (p*I* 8.11, 34 kDa), spot15 (p*I* 9.28, 34 kDa) and spot 16 (p*I* 9.54, 34 kDa) were significantly elevated in *O. meyeriana*. These results were consistent with the SDS-PAGE analyses of glutelin acidic subunits in *O. meyeriana* (Figure 2A), which indicated that not only the protein spot numbers were elevated in glutelin acidic subunits of *O. meyeriana*, but also protein types were significantly different from those of other materials, indicating that there may be new protein types in glutelin acidic subunits of *O. meyeriana*.

Protein spots in the framed region increased noticeably in the three wild rice species (Additional file 1: Figure S1A-E), and a row of unique 32 kDa proteins appeared in *O. officinalis*, which also suggested that the expression of glutelin acidic subunits in wild rice species was higher than that in cultivated rice, and there may be new protein types. Moreover, the absent protein spots (spots 7, 13 and 14) and unique protein spots (spots 23, 24 and 25) in *O. officinalis*, whether these are mutual relationship between the two phenomena needs to be further studied.

### The expression of glutelin precursor (II) was higher in the three wild rice species than that of cultivated rice materials

The molecular weight of protein in glutelin precursor region was from 66 kDa to 45 kDa, and p*I* range was between 9.0 and 10.0. The protein expression in glutelin precursor of the three wild rice species was significantly higher than those of two cultivated rice (Additional file 2: Figure S2), revealing that quantity of glutelin precursor in wild rice species exceeded cultivated rice, especially of *O. rufipogon* and *O. meyeriana*, which was consistent with the result shown in Figure 2A.

### *O. meyeriana* had highest expression amount in glutelin basic subunits (III)

The molecular weight of protein in glutelin basic subunits was from 24 kDa to 20 kDa, and p*I* range was between 9.5 and 10.0. The expression amount of protein spot 19 (p*I* 9.95, 22 kDa) and spot 20 (p*I* 9.95, 23 kDa) in glutelin basic subunits were elevated in the three wild rice species in comparison to the two cultivated rice materials, especially those in *O. meyeriana* (Additional file 3: Figure S3). This indicated that the expression of glutelin basic subunits in the three wild rice species was higher than that of two cultivated rice materials, and *O. meyeriana* had the highest expression amounts in glutelin basic subunits. This can also be seen from the SDS-PAGE analysis of glutelin basic subunits in Figure 2A.

### There were two unique protein groups in water-soluble proteins (IV) in *O. meyeriana*

Four consecutive protein spots (Additional file 4: Figure S4A-E) with molecular weight 62 kDa and p*I* from 4.5 to 7.5 were elevated obviously in the three wild rice species comparing to the two cultivated rice materials. Accordingly, the expression of 62 kDa protein in wild rice species increased in comparison to cultivated rice materials, which was consistent with the result shown in the Figure 2A. In addition, compared with the rest of materials, we found there were two unique protein groups in *O. meyeriana*, as showed with the circle in Additional file 4: Figure S4E.

### A specific protein spot was present in globulins (V) in *O. officinalis*

Comparison of the globulin among five materials showed noticeable differences. Spot 17 (p*I* 5.44, 24 kDa) and spot 19 (p*I* 5.65, 24 kDa) displayed apparent up-regulation in *O. meyeriana*. But they were absent on the corresponding position on the 2-DE gel in *O. officinalis.* Meanwhile, a specific protein spot 39 (p*I* 6.27, 24 kD) was present in *O. officinalis* (Additional file 5: Figure S5).

### Identified of differential protein spots in wild rice species by MS/MS

There was significantly different in glutelin acidic subunits among five materials. Among differentially accumulated protein spots in this region, six obvious difference protein spots, including spots 21 and 22 in *O. rufipogon*, spots 11 and 23 in *O. officinalis*, spots 14 and 27 in *O. meyeriana* (Additional file 1: Figure S1C-E) were chose and analyzed by LC/ESI-MS/MS. Among the identified of six differential protein spots of the wild rice species, the specific protein spot 23 in *O. officinalis* represented glutelin type-A 2 precursor. And the other five protein spots were annotated as hypothetical proteins (Table 4).

**Table 4:** **Identities of six differential protein spots of the three wild rice species by LC/ESI-MS/MS**

**Species**	**Spot number**	**Protein name**	**Accession number**	**Theoretical pI/MW(Da)**	**Actual pI/MW(Da)**	**Number of identified peptides**
*O. rufipogon*	21	Hypothetical protein	BAD33512	8.21/29986.11	6.68/34000	2
	22	Hypothetical protein OsJ_003768	EAZ13943	9/29819.11	6.83/34000	2
*O. officinalis*	11	Hypothetical protein OsJ_008621	EAZ25138	6.49/23679.04	6.99/34000	2
	23	GLUA2_ORYSJ Glutelin type-A 2 precursor	P07730	8.93/56306.23	5.95/34000	1
*O. meyeriana*	14	Hypothetical protein	BAD33512	8.21/29986.11	8.11/34000	2
	27	Hypothetical protein OsJ_021482	EAZ37999	7.28/98083.5	6.90/37000	3

Protein spots exhibited substantial discrepancies between the theoretical and experimental values in MW and/or p*I*. Mass spectrometry (theoretical) data of a protein is from the peptide segment alignment, but the experimental data of a protein is often from product of the protein modification, or splicing in database. The experimental molecular and p*I* values of spot 23 identified in *O. officinalis* by mass spectrometry was 34 kDa/5.95, which is in the glutelin type-A subunits range. Therefore, the specific protein spot 23 in *O. officinalis* was a new subunit protein through different splicing patterns from glutelin type-A precursor. The other five differential spots from the wild rice species were not identified as the precise corresponding proteins, indicating that there must excited new splicing types of glutelin type-A precursor in wild rice species.

### Initial identification of differential protein spots in wild rice species

Through compared our protein 2-DE maps of the three wild rice species with the 2-DE database and the study of Xie et al. [12], we predicted the identities of some of the differential protein spots from the three wild rice species. The identification results from these spots ranking at top 19 spots were listed in Additional file 6: Table S1. Meanwhile, we speculated more information about protein spots in differential column among the three wild rice species (Tables 5 and 6).

**Table 5:** **The corresponding protein spots of five materials compared with the Xie et al. studied**

**Protein type**	**Corresponding spot number**	^*^ **Protein names**
Glutelin acidic subunit	14, 15, 16	Putative glutelin type-B 2 precursor
Glutelin preursor	5	Glutelin type I precursor
Soluble protein region	1, 2, 3, 4	Glycogen (starch) synthase
Globulin region	17, 18	Alpha-globulin

^*^Protein names were identified by Xie et al. [12] and listed in Additional file 6: Table S1.

**Table 6:** **The difference of SDS-PAGE and 2-DE map of wild rice species and identification of the corresponding protein spots among three wild rice species**

**Differential protein region**	**SDS-PAGE**	**2-DE map**	^*^ **2-DE map identification by Xie et al.** **[** 12 **]**	**LC/ESI-MS/MS identification of wild rice species**
I, Glutelin acidic subunit	Expression of protein from 35 kDa to 34 kDa in this area of *O. meyeriana* was significantly higher than that of other materials.	The expression of protein spot 14, spot 15 and spot 16 with molecular weight 34 kDa and p*I*8.0 to 9.5 obviously increased in *O. meyeriana.*	Corresponding to spots 15 and 16 in the corresponding region which were identified as the 56 kDa putative glutelin type-B 2 precursor	No
	*α*-1 subunit of glutelin acidic subunit was absent, while a new protein band of about 40 kDa appeared in *O. meyeriana.*	Specific protein spot 23 (34 kDa, p*I*5.95), spot 24 (34 kDa, p*I*5.72) and spot 25 (34 kDa, p*I*5.54) were observed in *O. officinalis*; Protein spot 7 (35 kDa, p*I*7.22), spot 13 (34 kDa, p*I*7.58) and spot 14 (34 kDa, p*I*8.11) were absent in *O. officinalis*;.	Corresponding to spot 14 in the corresponding region which was identified as the 56 kDa putative glutelin type-B 2 precursor.	Protein spot 14: hypothetical protein
		Two unique protein spots 21 (p*I*6.68,34 kDa) and 22 (p*I*6.83,34 kDa) were detected in *O. rufipogon*.	No corresponding spot in the corresponding area.	Protein spot 23: 56 kD glutelin type-A 2 precursor
II, Glutelin precursor	The expression of 57 kDa glutelin precursor increased in *O. rufipogon*.	The expression of protein in this region with 57 kDa, and p*I*9.0-10.0 increased in the three wild rice species	Corresponding to spot 5 in the corresponding region which was identified as 56 kDa glutelin type I precursor.	No
IV, Water-soluble protein	The expression contents of 62 kDa protein in wild rice species were higher than two cultivated rice materials.	Four consecutive protein spots 1, 2, 3 and 4 with 62 kDa and p*I* from 4.5 to 7.5 were elevated in water-soluble protein area of the three wild rice species.	Corresponding to spots 1, 2, 3 and 4 in the corresponding region which were identified as glycogen (starch) synthase.	No
		Two unique protein groups were present in *O. meyeriana.*	No corresponding spot in the corresponding area.	No
V, Globulin	The 26 kDa protein band was absent, and a new 28 kDa protein band appeared in *O. meyeriana*.	Protein spots 17 (p*I*5.44, 24 kDa) and 18 (p*I*5.65, 24 kDa) were absent in *O. officinalis*, meanwhile a protein spot 39 with 24 kDa and p*I*6.27 was present.	Corresponding to spots 17 and 18 in the corresponding region which were identified as 21 kDa α globulin, and the experimental protein molecular weight is 26 kDa.	No
	The 26 kDa protein band in *O. officinalis* decreased remarkably.	The expression of protein spot 18 notably higher than the other material in *O. meyeriana*.		

^*^The study of endosperm protein 2-DE map identification by Xie et al.

Compared 2-DE proteins profiles of the three wild rice species with the cultivate rice endosperms protein 2-DE image of Xie et al., we found some endosperms protein spots which can also be found in wild rice species. The 2-DE images of the three wild rice species were similar to endosperms protein 2-DE image of Xie et al., and there existed many corresponding spots between them. Major corresponding spots included spot 14, spot 15 and spot 16 in glutelin acidic subunits, spot 5 in glutelin precursor, spot 1, spot 2, spot 3 and spot 4 in water-soluble protein, and spot 17 and spot 18 in globulin in Figure 3, which were indicated with arrows and numbers.

Table 6 showed the comprehensive analyses of differentially expressed proteins in the three wild rice species through combined analyses of their SDS-PAGE, 2-DE maps, LC/ESI-MS/MS identification, and previously reported 2-DE maps and proteins identification [13]. Through analyzed from Table 6, we speculated that there maybe excite specific expression patterns of 56 kDa glutelin type-A 2 precursor, and maybe its alternative splicing cause glutelin differences in *O. officinalis*. In the three wild rice species, protein band with 62 kDa was up-regulated, which may be resulted from difference in starch synthase protein of 66 kDa, suggesting that the expression of starch synthase protein in wild rice species were higher than those of cultivated rice.

## Discussion

The main purpose to improve rice seed quality was to increase the contents of its lysine and total proteins. Glutelin and prolamine are two major storage proteins in rice seed. Glutelin contains higher amount of essential amino acid of lysine. And prolamine is generally rich in leucine, but poor in lysine and sulfur-containing amino acids [14]. Rice seed proteins localize in two types of protein bodies (PB), PB-I and PB-II. PB-I contains prolamines, whereas PB-II is rich in glutelins and globulin [4,6]. Moreover, PB-I is less digestible than PB-II [15] suggesting that prolamine in PB-I is nutritionally less meaningful for human consumption than glutelin in PB-II. Therefore, the nutritional value of rice could thus be enhanced by increasing its easily digested glutelin and globulin contents, and meanwhile reducing prolamine contents [7].

Through the determination of seed protein contents in the three wild rice species and two cultivated rice materials, we found that different glutelin contents mainly resulted in different total seed protein contents. Comparisons of SDS-PAGE and 2-DE maps among five materials (Figures 2A and 3) showed that in terms of protein profiles, *O. rufipogon* was similar to the two cultivated rice, especially very close to *O. sativa Indica* Dianlong201; however, *O. officinalis* and *O. meyeriana* (especially of *O. meyeriana* ) showed significantly different from the two cultivated rice. This is also consistent with the traditional genetic difference among them.

By compared with the study of Xie et al. [12], we found that the protein expression in wild rice species were significantly higher than those in cultivated rice as follows: the 66 kDa starch synthase protein, 56 kDa glutelin type-Iprecursor and putative 56 kDa glutelin type-B 2 precursors. The main differential proteins among three wild rice species were glutelin and glutelin precursor in glutelin acidic subunits (I) (Additional file 1: Figure S1). There existed many new protein spots of glutelin in wild rice species, which could provide the materials for improving rice quality.

The follow-up analysis and identification of total seed proteins in the wild rice species indicated that glutelin and glutelin precursor exhibited the greatest changes among seed proteins, and among them the main quality-related proteins in the three wild rice species from Yunnan province are as follows:

### The 56 kDa glutelin type-B 2 precursor

Different splicing or post-translational modifications patterns of putative 56 kDa glutelin type-B 2 precursor resulted in elevated expression of some protein spots in glutelin acidic subunits region with 34 kDa in wild rice species. While in *O. meyeriana*, there may be strong up-regulation factors which caused the strong expression of type-B 2 precursor, or there may be new similar glutelin precursor. The expression of spot 14 in *O. meyeriana* was significantly high, which was identified by Xie et al. [12] as the 56 kDa putative glutelin type-B 2 precursor. However, spot 14 in *O. meyeriana* was identified as hypothetical protein by LC-MS/MS, which did not match the protein reported by Xie et al., indicating that there might exist new types of glutelin in *O. meyeriana*.

### The 56 kDa glutelin type-I precursor

The higher expression of 56 kDa glutelin precursor type-I might result in elevated expression of proteins in 57 kDa glutelin precursor of *O. rufipogon*. But we are not completely sure of which due to the lack of mass spectrometric identification. While we can initially speculate that the proteins belong to the glutelin precursor family or a new glutelin precursor. In the same region, there maybe another new increasing glutelin precursor that causes the elevated expression of proteins in *O. meyeriana*.

### The 56 kDa glutelin type-A 2 precursor

In *O. officinalis*, specific protein spots 23, 24 and 25 were simultaneously present, while spots 7, 13 and 14 were absent in glutelin acidic subunits (Additional file 1: Figure S1D). The specific protein spot 23 was identified as glutelin type-A 2 precursor in *O. officinalis*, suggesting that it was formed through alternative splicing and modification of glutelin type-A 2 precursor. It was not observed on 2-DE maps of the other materials, indicating that this was a unique splicing and modification pattern of type-A 2 precursor in *O. officinalis*, which was different from that of the other materials. During glutelin formation, glutelin precursor is processed into acidic and basic subunits by post-translational cleavage [4,5]. The same glutelin precursor may be processed into different protein subunits through different cleavage patterns. We can infer that glutelin type-A 2 precursor was processed into protein subunit of three spots 7, 13 and 14 through cleavage in cultivated rice, while it was processed into protein subunits of three spots 23, 24 and 25 through a novel cleavage way in *O. officinalis*.

### Other proteins

Previous study found that the 26 kDa globulin was deposited into PB- II with glutelin [6,16]. There was lack of 26 kDa globulin or with a highly reduced 26 kDa globulin contents in *O. meyeriana* and *O. officinalis* (Figure 2A and Additional file 5: Figure S5D and E). The 26 kDa globulin reduction accompanied accumulation of high levels of free amino acids in rice grains [17]. Free amino acids in rice are the ingredients to improve palatability. There may be high contents of free amino acids in *O. meyeriana* and *O. officinalis*, which can open a new research direction for improving rice nutritional quality and palatability. In the present study, the presence of glutelin and prolamine in three wild rice varieties could be further exploited for high contents of proteins and amino acids.

The expression of glutelin precursor was higher in three wild rice species than cultivated rice, and there existed many new types of glutelin subunits. These wild rice species can be used to improve rice quality. Up-regulation of glutelin precursor similar to that of cultivated rice may exist in *O. rufipogon*, from which we can seek regulatory elements of the relevant genes. *O. officinalis* and *O. meyeriana* had low contents of 26 kDa globulin or even lack this protein bands, indicated they maybe possess a new splicing pattern of glutelin precursor. Cheng et al. [10] determined the contents of 17 amino acids in husked seeds of the three wild rice species and six cultivated rice which were collected from similar regions, and found *O. officinalis* contained the highest amount of 17 amino acids, seven of which analyzed were essential amino acids for human boby. These will lead us to find the reasons for high free amino acids in *O. officinalis*. Meanwhile, in *O. meyeriana*, with high contents of glutelin and a relatively low contents of prolamine, in which there maybe exist many new types of glutetin and glutetin precursors. Many rice mutants by mutagenesis had low glutelin and high prolamine contents [7,18-20], while few rice materials with high glutelin and low prolamine contents have been obtained so far. Hence, *O. meyeriana* and *O. officinalis* having high glutelin contents and low prolamine contents can be suggested as natural genetic resource for improving rice glutelin contents of rice seed.

Some differential proteins among wild rice species were not identified through LC/ESI-MS/MS searching against NCBI *Oryza sativa japonica*, although they were well identified in the Maize database. According to previous 2-DE studies of rice seed proteins, they should belong to the storage proteins. This also indicated that some new and completely different types of glutelin or glutelin precursors exist in the three wild rice species.

## Methods

### Extraction of total seed storage proteins

Mature and plump seeds of the three wild rice species (*O. rufipogon*, *O. officinalis* and *O. meyeriana*) and two cultivated rice materials (*O. sativa Japonica* Hexi 35 and *O. sativa Indica* Dianlong 201) were collected from the field of Jinghong in the Yunnan province of China. Seeds were dehulled, crushed with a hammer, and quickly poured into a mortar, then ground into fine powder with pestle in liquid nitrogen. For the total protein extraction, 60 mg powder of each material was respectively homogenized with 1 ml of lysis buffer (7 M Urea, 2 M Thiourea, 4% (w/v) CHAPS, 2% (v/v) pH 3-10 IPG buffer, 40 mM DTT, 10 mM PMSF). The homogenates were vortexed vigorously and incubated at room temperature for 2 hr in shaker (HZ-9211 KB, Hualida Labs, Taicang, China), and then centrifuged at room temperature at 17800 g for 20 min (eppendrorf centrifuge 5417R, Gene company limited) until the supernatant was clear. The extracted storage protein samples were collected as the final supernatant and stored at -80°C until use.

### Fractionation of proteins

Albumin-globulin, prolamine and glutelin were extracted sequentially according to Kumamaru et al. [7] and Iida et al. [18] with minor modifications. 200 mg powder of each sample was respectively transferred into 1.5 ml centrifugation tubes and suspended in 1 ml solution A (10 mM Tris-HCl, pH 6.8, 0.5 M NaCl) and shaken vigorously. The suspension was incubated at room temperature for 6 hr in a shaker (HZ-9211 KB), vortexes vigorously every 30 min, and then centrifuged at 4°C at 13100 g for 15 min (eppendrorf 5417R). Albumin and globulin were collected as supernatant. And this step was repeated once. The precipitate was suspended with 1 ml solution B (60% n-propanol containing 5% 2-mercaptoethanol) for extraction of prolamine and the extraction procedure was done as above. Finally, the previous precipitate was suspended in 1 ml solution C (1% lactic acid containing 1 mmol/L EDTA-2Na) for extraction of glutelin in the same way as the extraction of prolamine. All supernatants were stored at -80°C.

### Protein determination

The protein concentration was determined on the basis of the bovine serum albumin (BSA) using the Bradford assay [21]. Protein content of the original sample (mg/ml)= dilution multiple × actual value (μg)/10 μl.

### 1-D SDS-PAGE and 2-DE analysis

For 1-D SDS-PAGE, 15 μg of protein extraction was loaded into each well and then separated on 12% polyacrylamide gel (acrylamide to bis acrylamide ratio 29:1) and 5% (w/v) stacking gel. Proteins were visualized by staining overnight with Coomassie Brilliant Blue R-250(CBB R-250, Ameressco, Solon, OH, USA). For 2-DE, 150 μl of protein sample was mixed with 150 μl of rehydration buffer (7 M Urea, 2 M Thiourea, 20 mM DTT, 0.002% bromophenol blue) and centrifuged at room temperature at 14000 rpm for 5 min. The pH 3-10 IPG strips (13 cm, GE Healthcare, USA) were rehydrated for 12 hr at 20°C in 250 μl of rehydration buffer containing protein samples. After which isoelectric focusing (IEF) was performed for a total of 35000 V. hr with 50 μA per strip using IPGPhor II (GE Healthcare) at 20°C. Equilibration of the strips was incubated on an orbital shaker for 15 min in 10 ml equilibration buffer (6 M Urea, 75 mM Tris-HCl, pH 8.8, 29.3% v/v glycerol, 2% w/v SDS, 0.002% bromophenol blue) containing 1% w/v DTT, followed by 15 min in 10 ml equilibration buffer containing 2.5% iodoacetamide. Afterwards, the strips were placed on the top of vertical 12.5% polyacrylamide gels and sealed with 1% low-melting agarose solution. SDS-PAGE was run on an Ettan DALT six electrophoresis system (GE Healthcare) at 2 W/gel for 40 min and followed by 17 W/gel for 4 hr until the bromophenol blue tracking dye front reached about 1 cm from the bottom of the gel. 2-DE of five samples was performed at the same time to minimize experimental error, and at least 3 replicates were performed for each material.

### Gel staining and image analysis

After electrophoresis, the gels were stained with Blue Silver [22] using Coomassie Brilliant Blue G-250 (CBB G-250, Ameressco, Solon, OH, USA). Gels were rinsed with MilliQ water (Millipore, Bedford, MA, USA) for 30 s, fixed in a solution of 10% acetic acid and 40% ethanol for 2 × 30 min. Then gels were stained overnight on an orbital shaker in a CBB G-250(Ameressco, Solon, OH, USA) solution consisting of 0.12% G-250, 10% ammonium sulfate, 10% phosphoric acid and 20% methanol, and then distained with MilliQ water 3-4 times for 30 min each until no background. Gels were scanned at 300 dpi resolution using the ImageScanner and acquired with ImageMaster system.

Gel Image analyses were carried out with ImageMaster 2D platinum software (GE Healthcare) including image editing, protein spots detection, protein spots measurement, matching, image data analysis and integration. Spot detection was performed with the parameters saliency, smooth and minimum area set to 60, 2 and 50, respectively. Only those with significant and reproducible changes were considered to be different proteins. Experimental molecular weight (MW) values of spots were calculated in comparison with protein standard marker run on the gel, and their p*I* values were calculated by the liner pH arrangement of IPG strips (GE Healthcare).

### MS and data analysis

Protein spots showed apparent variation between wild rice and cultivated rice were excised manually from the CBB-stained gels. LC/ESI-MS/MS analysis was done by Institute of Protein Research & Analysis Center, Shanghai Institutes for Biological Sciences Biochemistry and Cell Biology of the Chinese Academy of Sciences, which provide us with the specific methods and procedure of protein identification. The proteins were digested in-gel using trypsin. The eluted, trypsin-generated peptides were subsequently separated by a Capillary High Performance Liquid Chromatography (HPLC) system (LCQ, ThermoFinnigan, San Jose, CA). Mobile phase A consisted of 0.1% (v/v) formic acid, while mobile phase B was composed of 0.1% (v/v) formic acid in 84% (v/v) acetonitrile. Subsequently, ESI-MS/MS were performed on a Surveyor LCQ Deca IT mass spectrometer (ThermoFinnigan, San Jose,CA). Acquired raw data from ESI-MS/MS analysis were processed using Sequest algorithm at Bioworks 3.2 against NCBI *Oryza sativa japonica*. Filtered criterion was as follows: Xcorr ≥ 1.9, 2.2 and 3.75 for charge 1+, 2+, and 3+, DelCN ≥ 0.1.

## Additional files

Additional file 1: Figure S1. The protein spots of glutelin acidic subunits among five rice materials. The compared protein spots were indicated with the corresponding numbers and arrows, at which some noticeably increased spots in three wild rice species were framed. (A) *O. sativa japonica* Hexi35; (B) *O. sativa indica* Dianlong201; (C) *O. rufipogon*; (D) *O. officinalis*; (E) *O. meyeriana.*
Additional file 2: Figure S2. The expression of glutelin precursors is higher in wild rice species than that of cultivated rice. The circles indicated the difference of protein expression in five materials. (A) *O. sativa japonica* Hexi35; (B) *O. sativa indica* Dianlong201; (C) *O. rufipogon*; (D) *O. officinalis*; (E) *O. meyeriana.*
Additional file 3: Figure S3. Comparison of glutelin basic subunits among five materials. The protein expression of corresponding protein spots, indicated with arrows and numbers, were higher in wild rice species than that of cultivated rice. (A) *O. sativa japonica* Hexi35; (B) *O. sativa indica* Dianlong201; (C) *O. rufipogon*; (D) *O. officinalis*; (E) *O. meyeriana.*
Additional file 4: Figure S4. Two unique groups of proteins were present in water-soluble protein in *O. meyeriana*. The expression of four protein spots, indicated with arrows and numbers, were higher in wild rice species than that of cultivate rice. (A) *O. sativa japonica* Hexi35; (B) *O. sativa indica* Dianlong201; (C) *O. rufipogon*; (D) *O. officinalis*; (E) *O. meyeriana.*
Additional file 5: Figure S5. Comparison of globulins among five materials. The reference protein spots indicated with arrows and numbers. There were no protein spots 17 and 18, and a specific protein spot was present in *O. officinalis*. (A) *O. sativa japonica* Hexi35; (B) *O. sativa indica* Dianlong201; (C) *O. rufipogon*; (D) *O. Officinalis*; (E) *O. meyeriana.*
Additional file 6: Table S1. The 19 endosperm protein spots of three cultivated rice which are shared by five materials.

## Abbreviations

1-D SDS-PAGE: One-dimensional SDS- polyacrylamide gelelectrophoresis
2-DE: Two-dimensional polyacrylamide gel electrophoresis
SDS: Sodium dodecyl sulfate
CBB: Coomassie Brilliant Blue
LC/ESI-MS/MS: Liquid chromatography/electrospray ionization-tandem mass spectrometry
CHAPS: 3-[(3-cholamidopropyl)dimethylamonio]-1-propanesulfonate
DTT: Dithiothreitol
PMSF: Phenylmethylsulfonyl fluoride
Xcorr: Cross-correlation score
DeltaCn: Normalized difference in correlation score
