# Supplementary material for: Proteomic analysis of seed storage proteins in wild rice species of the Oryza genus
Source: Proteome Sci. 2014 Nov 30;12:51. doi: 10.1186/s12953-014-0051-4 (PMC4263040; doi:10.1186/s12953-014-0051-4)
Supplement: Additional file 1: Figure S1. — The protein spots of glutelin acidic subunits among five rice materials. The compared protein spots were indicated with the corresponding numbers and arrows, at which some noticeably increased spots in three wild rice species were framed. (A) O. sativa japonica Hexi35; (B) O. sativa indica Dianlong201; (C) O. rufipogon; (D) O. officinalis; (E) O. meyeriana. [file 12953_2014_51_MOESM1_ESM.doc]

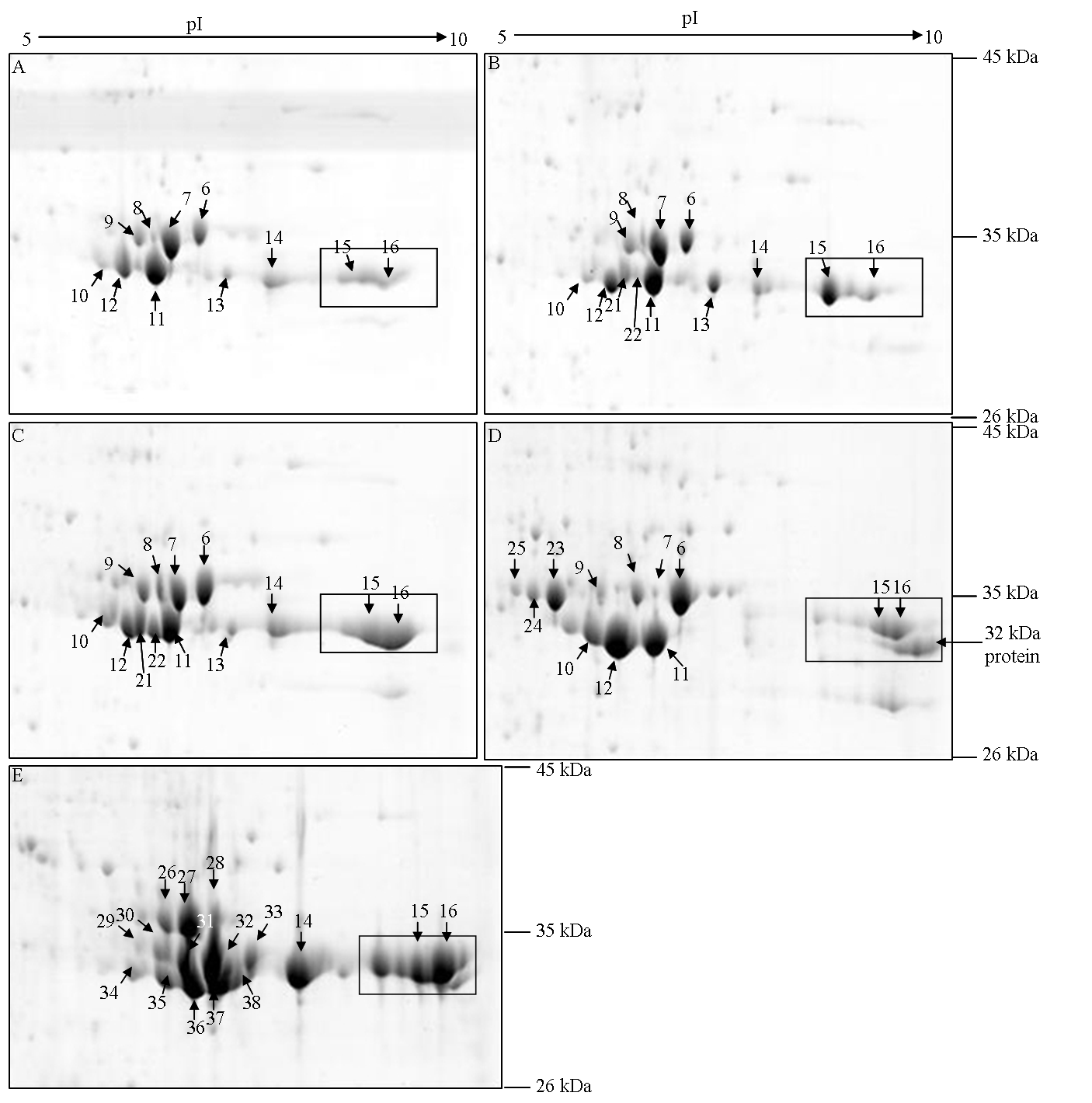


**Additional file 1: Figure S1. The protein spots of glutelin acidic subunits among five rice materials.** The compared protein spots were indicated with the corresponding numbers and arrows, at which some noticeably increased spots in three wild rice species were framed. (A) *O. sativa japonica* Hexi35; (B) *O. sativa indica* Dianlong201; (C) *O. rufipogon*; (D) *O. officinalis*; (E) *O. meyeriana.*
