# Supplementary material for: Proteomic analysis of seed storage proteins in wild rice species of the Oryza genus
Source: Proteome Sci. 2014 Nov 30;12:51. doi: 10.1186/s12953-014-0051-4 (PMC4263040; doi:10.1186/s12953-014-0051-4)
Supplement: Additional file 2: Figure S2. — The expression of glutelin precursors is higher in wild rice species than that of cultivated rice. The circles indicated the difference of protein expression in five materials. (A) O. sativa japonica Hexi35; (B) O. sativa indica Dianlong201; (C) O. rufipogon; (D) O. officinalis; (E) O. meyeriana. [file 12953_2014_51_MOESM2_ESM.doc]

**
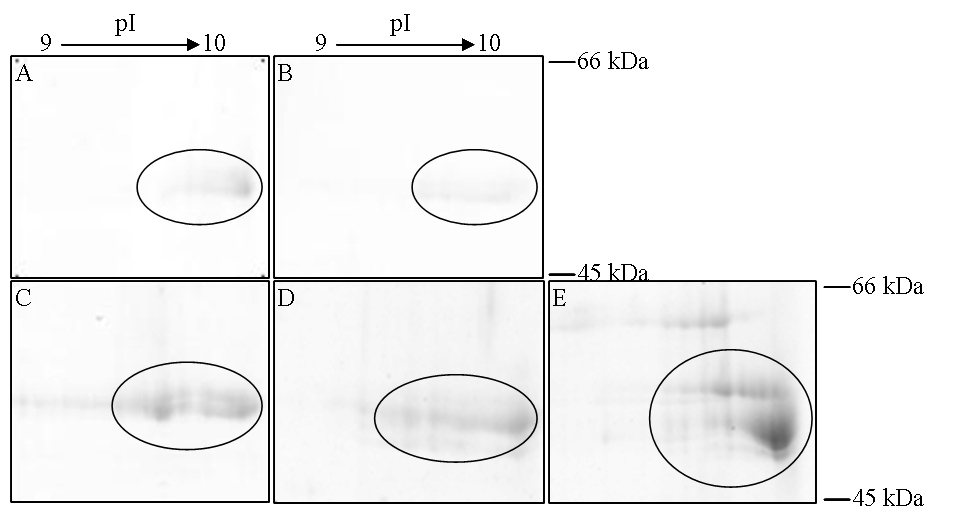
**

**Additional file 2: Figure S2 The expression of glutelin precursors is higher in wild rice species than that of cultivated rice.** The circles indicated the difference of protein expression in five materials. (A) *O. sativa japonica* Hexi35; (B) *O. sativa indica* Dianlong201; (C) *O. rufipogon*; (D) *O. officinalis*; (E) *O. meyeriana.*
