# Supplementary material for: Proteomic analysis of seed storage proteins in wild rice species of the Oryza genus
Source: Proteome Sci. 2014 Nov 30;12:51. doi: 10.1186/s12953-014-0051-4 (PMC4263040; doi:10.1186/s12953-014-0051-4)
Supplement: Additional file 3: Figure S3. — Comparison of glutelin basic subunits among five materials. The protein expression of corresponding protein spots, indicated with arrows and numbers, were higher in wild rice species than that of cultivated rice. (A) O. sativa japonica Hexi35; (B) O. sativa indica Dianlong201; (C) O. rufipogon; (D) O. officinalis; (E) O. meyeriana. [file 12953_2014_51_MOESM3_ESM.doc]

**
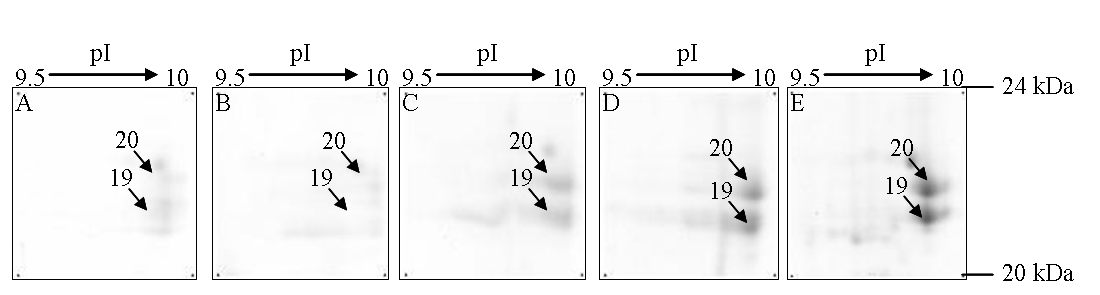
**

**Additional file 3: Figure S3. Comparison of glutelin basic subunits among five materials.** The protein expression of corresponding protein spots, indicated with arrows and numbers, were higher in wild rice species than that of cultivated rice. (A) *O. sativa japonica* Hexi35; (B) *O. sativa indica* Dianlong201; (C) *O. rufipogon*; (D) *O. officinalis*; (E) *O. meyeriana.*
