# Supplementary material for: Proteomic analysis of seed storage proteins in wild rice species of the Oryza genus
Source: Proteome Sci. 2014 Nov 30;12:51. doi: 10.1186/s12953-014-0051-4 (PMC4263040; doi:10.1186/s12953-014-0051-4)
Supplement: Additional file 4: Figure S4. — Two unique groups of proteins were present in water-soluble protein in O. meyeriana. The expression of four protein spots, indicated with arrows and numbers, were higher in wild rice species than that of cultivate rice. (A) O. sativa japonica Hexi35; (B) O. sativa indica Dianlong201; (C) O. rufipogon; (D) O. officinalis; (E) O. meyeriana. [file 12953_2014_51_MOESM4_ESM.doc]

**
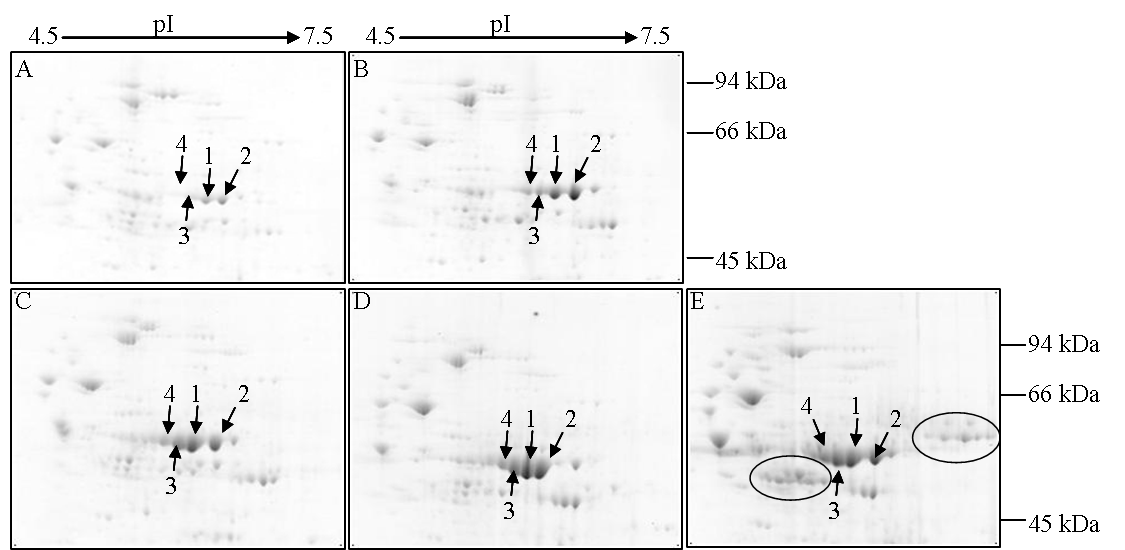
**

**Additional file 4: Figure S4. Two unique groups of proteins were present in water-soluble protein in *O. meyeriana*.** The expression of four protein spots, indicated with arrows and numbers, were higher in wild rice species than that of cultivate rice. (A) *O. sativa japonica* Hexi35; (B) *O. sativa indica* Dianlong201; (C) *O. rufipogon*; (D) *O. officinalis*; (E) *O. meyeriana.*
