# Supplementary material for: Proteomic analysis of seed storage proteins in wild rice species of the Oryza genus
Source: Proteome Sci. 2014 Nov 30;12:51. doi: 10.1186/s12953-014-0051-4 (PMC4263040; doi:10.1186/s12953-014-0051-4)
Supplement: Additional file 5: Figure S5. — Comparison of globulins among five materials. The reference protein spots indicated with arrows and numbers. There were no protein spots 17 and 18, and a specific protein spot was present in O. officinalis. (A) O. sativa japonica Hexi35; (B) O. sativa indica Dianlong201; (C) O. rufipogon; (D) O. Officinalis; (E) O. meyeriana. [file 12953_2014_51_MOESM5_ESM.doc]

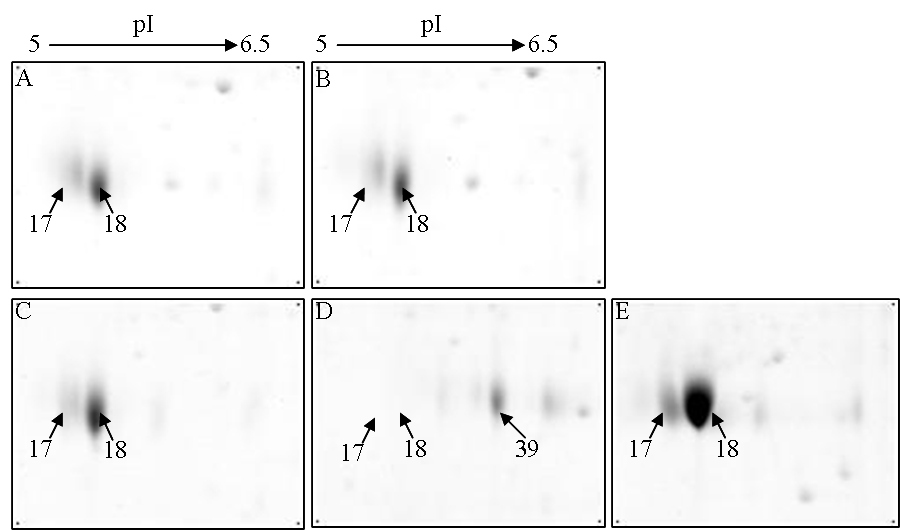


**Additional file 5: Figure S5. Comparison of globulins among five materials.** The reference protein spots indicated with arrows and numbers. There were no protein spots 17 and 18, and a specific protein spot was present in *O. officinalis*. (A) *O. sativa japonica* Hexi35; (B) *O. sativa indica* Dianlong201; (C) *O. rufipogon*; (D) *O. Officinalis*; (E) *O. meyeriana.*
