# Supplementary material for: Proteomic analysis of seed storage proteins in wild rice species of the Oryza genus
Source: Proteome Sci. 2014 Nov 30;12:51. doi: 10.1186/s12953-014-0051-4 (PMC4263040; doi:10.1186/s12953-014-0051-4)
Supplement: Additional file 6: Table S1. — The 19 endosperm protein spots of three cultivated rice which are shared by five materials. [file 12953_2014_51_MOESM6_ESM.doc]

**Additional file 6: Table S1. The 19 endosperm protein spots of three cultivated rice which are shared by five materials.**

| Spot  no. | GI no.^a)^ | Accession  no.^b)^ | pI Calc.c)/  Obs.^d)^ | MW Calc./  Obs. | Protein name |
| --- | --- | --- | --- | --- | --- |
| 1 | gi|297424 | CAA46294 | 8.34/6.698 | 66 994/63 452 | Glycogen (starch) synthase |
| 2 | gi|297424 | CAA46294 | 8.34/6.698 | 66 994/63 888 | Glycogen (starch) synthase |
| 3 | gi|297424 | CAA46294 | 8.34/6.698 | 66 994/63 019 | Glycogen (starch) synthase |
| 4 | gi|297424 | CAA46294 | 8.34/6.698 | 66 994/63 452 | Glycogen (starch) synthase |
| 5 | gi|50902034 | XP_463450 | 9.09/9.613 | 56 782/58 447 | Glutelin type I precursor |
| 6 | gi|31455453 | BAC77349 | 6.60/7.435 | 36 038/38 623 | Glutelin |
| 7 | gi|225710 | 1311273A | 8.93/7.103 | 56 727/38 491 | Glutelin |
| 8 | gi|225710 | 1311273A | 8.93/6.731 | 56 727/37 967 | Glutelin |
| 9 | gi|7436606 | S65073 | 8.35/6.464 | 39 141/38 097 | Fructose-bisphosphate aldolase |
| 10 | gi|100680 | D34332 | 8.81/6.124 | 56 390/37 451 | Glutelin 22 precursor – rice |
| 11 | gi|100680 | D34332 | 8.81/6.375 | 56 390/36 439 | Glutelin 22 precursor – rice |
| 12 | gi|100680 | D34332 | 8.81/6.593 | 56 390/36 066 | Glutelin 22 precursor – rice |
| 13 | gi|50907885 | XP_465431 | 8.96/7.759 | 57 426/36 815 | Glutelin |
| 14 | gi|50906685 | XP_464831 | 9.11/8.277 | 56 411/36 314 | Putative glutelin type-B 2 precursor |
| 15 | gi|50906685 | XP_464831 | 9.11/9.038 | 56 411/36 066 | Putative glutelin type-B 2 precursor |
| 16 | gi|50906685 | XP_464831 | 9.11/9.402 | 56 411/36 190 | Putative glutelin type-B 2 precursor |
| 17 | gi|51038053 | AAT93857 | 7.48/5.816 | 21 497/26 142 | Alpha-globulin |
| 18 | gi|51038053 | AAT93857 | 7.48/6.213 | 21 497/25 522 | Alpha-globulin |
| 19 | gi|34900098 | NP_911395 | 8.06/8.35 | 18 423/15 219 | Seed allergen RAG2 |

a) GI no. of NCBI database

b) Accession no. of NCBI database

c) Calc. is a value on the database

d) Obs. is a value on the experimen
